# Global Navigation Satellite System Real-Time Kinematic Positioning Framework for Precise Operation of a Swarm of Moving Vehicles

**DOI:** 10.3390/s22207939

**Published:** 2022-10-18

**Authors:** Euiho Kim, Sae-kyeol Kim

**Affiliations:** 1 Department of Mechanical & System Design Engineering, Hongik University, Seoul 04066, Korea; 2 Department of Mechanical Engineering, Hongik University, Seoul 04066, Korea

**Keywords:** global navigation satellite systems, RTK, relative position, multi agent operation

## Abstract

The global navigation satellite system (GNSS) real-time kinematic (RTK) technique is used to achieve relative positioning centimeter levels among multiple agents on the move. A typical GNSS RTK estimates the relative positions of multiple rover receivers with respect to a single-base receiver. In a fleet of rover GNSS receivers, this approach is inefficient because each rover receiver only uses GNSS measurements of its own and those sent from a single-base receiver. In this study, we propose a novel GNSS RTK framework that facilitates the precise positioning of a swarm of moving vehicles through the GNSS measurements of multiple receivers and broadcasts fixed-integer ambiguities of GNSS carrier phases. The proposed framework not only provides efficient RTK positioning but also reliable performance with a limited number of GNSS satellites in view. Our experimental flight tests with six GNSS receivers showed that the systematic procedure of the proposed framework could maintain lower than 6 cm of 3D RMS positioning errors, whereas the conventional RTK failed to resolve the correct integer ambiguities of double difference carrier phase measurements more than 13% in five out of nine total baselines.

## 1. Introduction

Real-time kinematic (RTK) positioning of global navigation satellite systems (GNSS) have been widely used for the realization of precise relative positions [1,2,3]. The RTK system can achieve centimeter-level positioning by processing carrier phase measurements as ranging sources. The RTK process includes integer ambiguity resolution through various algorithms, such as the least-squares ambiguity decorrelation adjustment (LAMBDA) [4,5]. The success probability of correctly resolved integer ambiguities of LAMBDA improves as the number of GNSS constellations and measurement frequencies increases [6,7,8,9]. Another approach to further improve the probability of a correct integer fix is to use known baseline information between the reference and rover GNSS receivers, which is commonly incorporated in GNSS-based attitude determination problems [10,11].

For the interoperability of RTK among different GNSS receiver manufacturers, the required GNSS measurements and satellite information for a rover to compute RTK solutions were established by the Radio Technical Commission Maritime Services (RTCM) Special Committee-104 (SC-104). The RTCM1040 v2.3 standards were used for a conventional RTK process between a pair of rovers and reference receivers. The RTCM 1040 v2.3 standards had 64 types of messages, of which, 1 through 17 messages were defined for a differential GNSS process, whereas messages 18 to 21 were added for future RTK solutions. The added messages included uncorrected carrier phase measurements, uncorrected pseudorange measurements, RTK carrier phase corrections, and RTK pseudorange corrections [12]. Later, RTCM 1040 v3 was released to support the operation of an RTK network with higher efficiency in terms of the broadcast bandwidth and higher integrity [13,14,15]. Currently, various network RTK methods, such as virtual reference stations, FKP, and the master-auxiliary concept, use RTCM standards as message containers.

More recently, the RTK method has been extended to use multiple reference receivers to further improve the reliability of the integer ambiguity resolution [16,17,18,19]. Wu et al. proposed a least-squares-based antenna array-aided RTK using more than one static GNSS receiver whose coordinates were accurately surveyed [16,17]. The authors in [18] introduced antenna array-aided precise point positioning (A-PPP), wherein integer ambiguities were resolved by an orthonormality-constrained multivariate mixed-integer least-squares method. The principles of the A-PPP were further enhanced to speed up the resolution of the integer ambiguities in continuously operating reference stations [19]. In these approaches, the known baselines between multiple base stations and corresponding double difference (DD) integer ambiguities effectively increase the number of measurements, such that a user can compute RTK solutions more reliably using LAMBDA [20,21]. Luo et al. presented an alternative approach for the relative positioning of multiple moving platforms at the centimeter level, which searched for DD integer ambiguities using the constraints imposed on the moving platforms rather than using LAMBDA [22,23].

**Figure 1:**
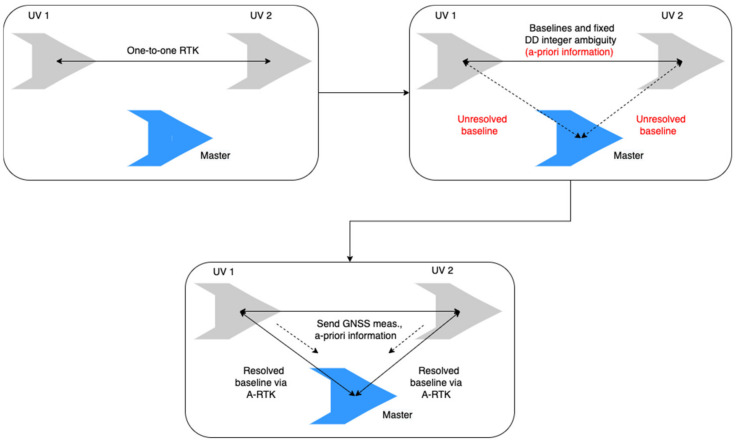
RTK positioning procedures in a cell consisting of two follower UVs and a master UV. Two UVs perform the one-to-one RTK first, and the estimated baselines and resolved integer ambiguities are sent to a master that implements antenna array-aided RTK (A-RTK).

**Figure 2:**
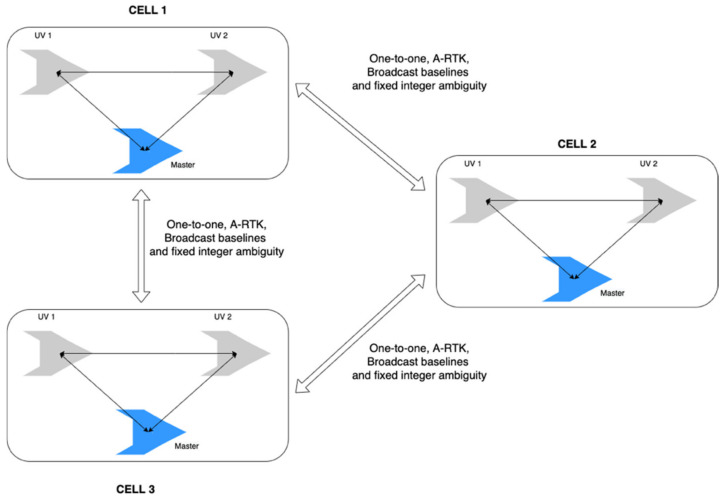
RTK process among three cells realized by the one-to-one RTK or antenna array-aided RTK (A-RTK) between the masters in the cells. The baselines among the UVs across the cells are computed with a broadcast of resolved fixed-integer ambiguities.

The antenna array-aided RTK is a powerful enabler for the operation of a swarm of unmanned vehicles (UV), wherein the precise relative position among UVs should be ensured in a reliable and fast manner. The communication channels, onboard GNSS receivers, and computational capability typically realized in a group of UVs can be used to implement an antenna array-aided RTK method without difficulty. Although the RTK process between a rover and base is well-established and commercialized, as observed in the RTCM standards, there have been limited discussions on the relative positioning of a swarm of UVs. This study introduces a framework that provides a systematic process for the relative positions of a group of UVs using an antenna array-aided RTK and the conventional RTK between a rover and a base, which is referred to as a one-to-one RTK. In the proposed framework, a swarm of UVs is composed of cells that consist of one master and up to two follower UVs. The relative positioning in the cell is computed using the two RTK methods, as shown in Figure 1, whereas the relative positioning across the UVs between different cells is accomplished in two steps. The first step is a one-to-one or antenna array-aided RTK process to resolve the DD integer ambiguities and baseline between the masters. The two masters exchange and broadcast the resolved DD integer ambiguities of other UVs in another cell to the UVs in their own cell, as illustrated in Figure 2. The broadcast fixed-integer ambiguities in a group of UVs are further processed to resolve the unfixed integer ambiguities between a pair of UVs without using search algorithms, such as LAMBDA. This study shows that the combination of RTK algorithms and broadcast-resolved or fixed-integer ambiguities among UVs is an effective and efficient solution for the relative positioning of multiple UVs, which would be useful for future RTCM standards for a swarm of moving vehicles.

The remainder of this paper is organized as follows. Section 2 presents an overview of antenna array-aided RTK techniques and introduces the integer ambiguity resolution strategy with broadcast fixed-integer ambiguities. Section 3 introduces a systematic procedure for determining the relative position of a swarm of UVs using a cell structure. Section 4 presents the experimental test results and conclusions are presented in Section 5.

## 2. Precise Positioning Using Antenna Array-Aided RTK and Broadcast Fixed-Integer Ambiguities

This section discusses the measurement formulation of an antenna array-aided RTK with three UVs. Subsequently, a systematic integer ambiguity resolution process with partially known fixed-integer ambiguities among the six UVs is discussed.

### 2.1. Measurement Formulation of Antenna Array-Aided RTK

**Figure 3:**
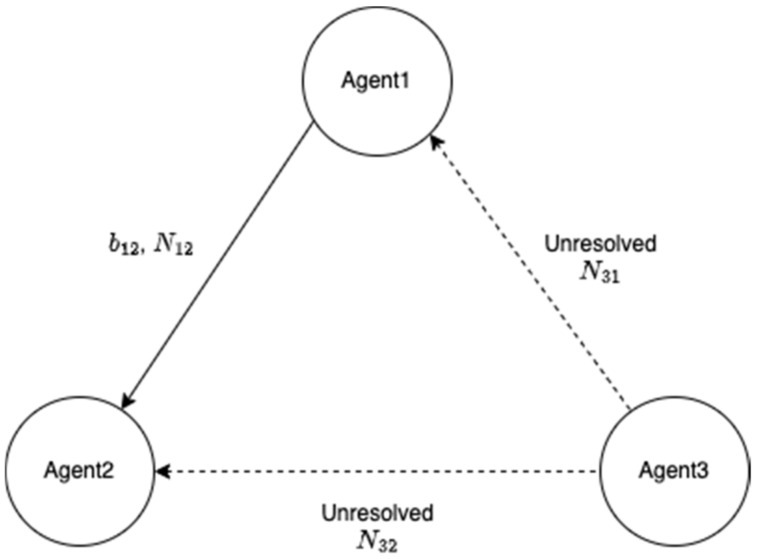
There are three agents, wherein agent 1 and agent 2 have correctly resolved integer ambiguities. The rest of the integer ambiguities need to be resolved.

Figure 3 shows three UVs, referred to as agents, and the baseline between agent 1 and agent 2 is assumed to be obtained with correctly resolved integer ambiguities using a one-to-one RTK. The DD code and carrier phase measurements among the three agents can be formulated as follows

(1) $$P_{12}=Gb_{12}+\varepsilon_{P,12}\;P_{31}=Gb_{31}+\varepsilon_{P,31}\;P_{32}=Gb_{32}+\varepsilon_{P,32}\;\Phi_{12}=\Lambda N_{12}+Gb_{12}+\varepsilon_{\Phi ,12}\;\Phi_{31}=\Lambda N_{31}+Gb_{31}+\varepsilon_{\Phi ,31}\;\Phi_{32}=\Lambda N_{32}+Gb_{32}+\varepsilon_{\Phi ,32}$$

where P is the DD pseudorange measurement. Φ is a DD carrier phase measurement. Λ is a diagonal matrix with a wavelength corresponding to a DD integer ambiguity vector, N. G is a DD line-of-sight matrix and b is a baseline vector. $\varepsilon_{P}$ and $\varepsilon_{\Phi }$ include the multipath and noise in the pseudo range and carrier phase measurements, respectively. Subscript ( · ) denotes a pair of agents in the DD measurements. Here, the two DD measurements were constructed using a common set of satellites and frequencies such that they had the same Λ and G.

Using the relationship between the baseline vectors and DD integer ambiguities, $P_{32}$ and $\Phi_{32}$ in Equation (1) can be expressed as follows:

(2) $$\begin{array}{cc} P_{32} & =Gb_{32}+\varepsilon_{P,32}\\ & =G\left(b_{31}+b_{12}\right)+\varepsilon_{P,32}\\ \Phi_{32} & =\Lambda N_{32}+Gb_{32}+\varepsilon_{\Phi ,32}\\ & =\Lambda \left(N_{3}-N_{2}+N_{1}-N_{1}\right)+G\left(b_{31}+b_{12}\right)\\ & =\Lambda \left(N_{31}-N_{12}\right)+G\left(b_{31}+b_{12}\right) \end{array}\;$$

We assume that the RTK solutions of $b_{12}$ and $N_{12}$, denoted as ${\overset{ˇ}{b}}_{12}$ and ${\overset{ˇ}{N}}_{12}$, respectively, are available as priori information. With this, a set of linear equations can be constructed from Equation (2) to estimate $b_{31}$ and $N_{31}$ as follows:

(3) $$\underset{Y}{\underset{︸}{\left[\begin{array}{c} \begin{array}{c} P_{32}-G{\overset{˘}{b}}_{12}\\ P_{31} \end{array}\\ \begin{array}{c} \Phi_{32}+\Lambda {\overset{˘}{N}}_{12}-G{\overset{˘}{b}}_{12}\\ \Phi_{31} \end{array} \end{array}\right]}}\;=\underset{A}{\underset{︸}{\left[\begin{array}{c} \begin{array}{c} \begin{array}{cc} G & 0 \end{array}\\ \begin{array}{cc} G & 0 \end{array} \end{array}\\ \begin{array}{c} \begin{array}{cc} G & \Lambda \end{array}\\ \begin{array}{cc} G & \Lambda \end{array} \end{array} \end{array}\right]}}\underset{X}{\underset{︸}{\left[\begin{array}{c} b_{31}\\ N_{31} \end{array}\right]}}+\left[\begin{array}{c} \begin{array}{c} \varepsilon_{P,32}\\ \varepsilon_{P,31} \end{array}\\ \begin{array}{c} \varepsilon_{\Phi ,32}\\ \varepsilon_{\Phi ,31} \end{array} \end{array}\right]$$

The float solutions of $b_{31}$ and $N_{31}$ and the covariance matrix can be computed from using weighted least-squares as follows

(4) $$\hat{X}=\left[\begin{array}{c} {\hat{b}}_{31}\\ {\hat{N}}_{31} \end{array}\right]={\left(A^{T}WA\right)}^{-1}A^{T}WY\;Cov\left(\hat{X}\right)=\left[\begin{array}{cc} Q_{{\hat{b}}_{31}} & Q_{{\hat{b}}_{31}{\hat{N}}_{31}}\\ Q_{{\hat{b}}_{31}{\hat{N}}_{31}}^{T} & Q_{{\hat{N}}_{31}} \end{array}\right]={\left(A^{T}WA\right)}^{-1}$$

where W is a DD weighting matrix and $Q_{\left(\;\cdot \;\right)}$ is a partial covariance matrix. With ${\hat{N}}_{31}$ and $Q_{{\hat{N}}_{31}}$, LAMBDA searches for a fixed integer solution denoted as ${\overset{˘}{N}}_{31}$ [4,5]. Then, the fixed baseline is computed as follows

(5) $${\overset{˘}{b}}_{31}={\hat{b}}_{31}-Q_{{\hat{b}}_{31}{\hat{N}}_{31}}Q_{{\hat{N}}_{31}}^{-1}\left({\hat{N}}_{31}-{\overset{˘}{N}}_{31}\right)$$

**Figure 4:**
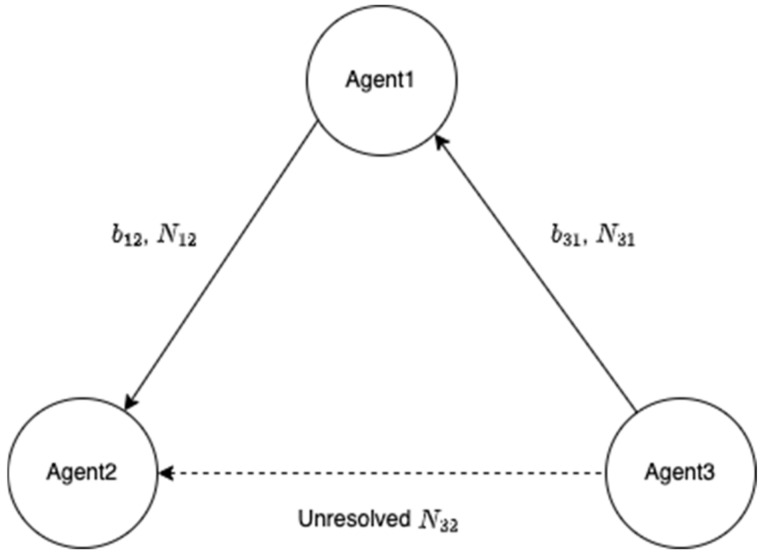
Updated baselines and DD integer ambiguities after implementing an antenna array-aided RTK.

When the fixed solution ${\overset{˘}{N}}_{31}$ is obtained, Figure 3 is updated to Figure 4. The remaining $N_{32}$ can then be computed with ${\overset{˘}{N}}_{12}$ and ${\overset{˘}{N}}_{31}$ by using the following relationship

(6) $$N_{32}=N_{3}-N_{2}\;\;\;\;\;\;\;\;\;=\left(N_{3}-N_{1}\right)-\left(N_{2}-N_{1}\right)\;\;\;\;\;\;\;\;=N_{31}-N_{21}$$

where $N_{2}$ and $N_{3}$ are the vectors of the single-difference integer ambiguities of the carrier phase measurements between the satellites in agents 2 and 3, respectively. It should be noted that ${\overset{˘}{N}}_{21}=-{\overset{˘}{N}}_{12}$.

Although Equation (6) is a straightforward computation, it has important practical advantages. First, $N_{32}$ can be computed from ${\overset{˘}{N}}_{12}$ and ${\overset{˘}{N}}_{31}$ without using LAMBDA, which would reduce the onboard computational resources. Additionally, reliable $N_{32}$ can be obtained even if the number of common satellites in view between agents 2 and 3 is small, such that the LAMBDA process may have a low chance of correctly resolving $N_{32}$.

### 2.2. Systematic Procedure for the Use of Broadcast Fixed-Integer Ambiguities

This subsection discusses how the broadcast fixed baseline and DD integer ambiguities of some UV pairs can be used to resolve DD integer ambiguities for other UVs in a group of UVs. For the explanation of the proposed method, we consider six agents whose fixed baselines and DD integer ambiguities have been determined in an unstructured way as a general case.

**Figure 5:**
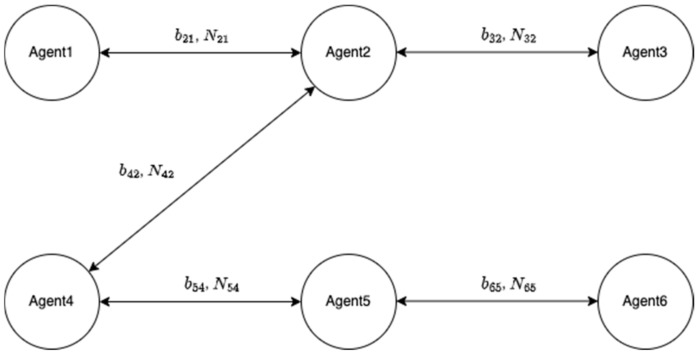
There are six agents in a group of UVs wherein five baselines and DD integer ambiguities are assumed to be correctly determined.

**Figure 6:**
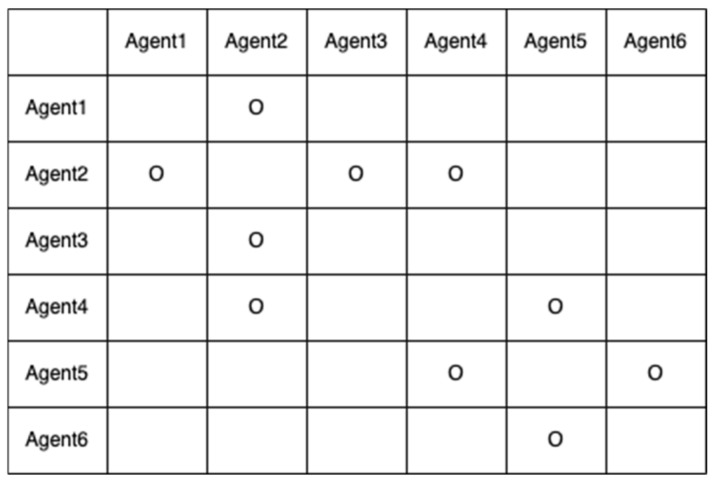
Integer ambiguity resolution table for the six agents in Figure 5.

**Figure 7:**
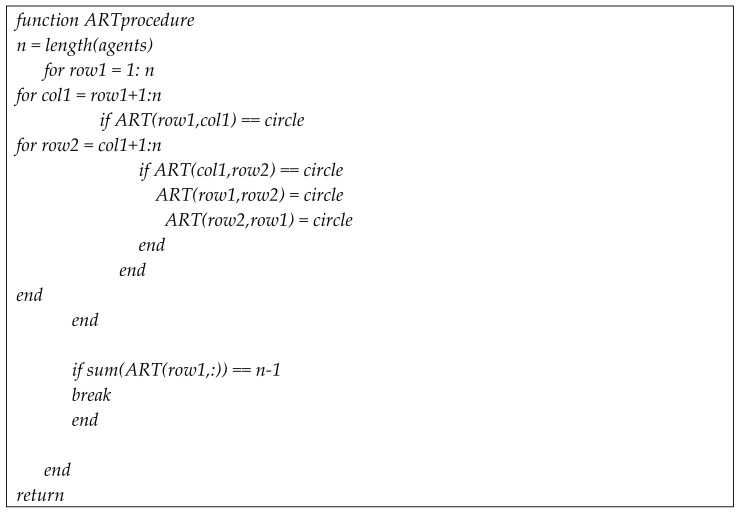
Pseudocode for the integer ART process.

Let us assume that their five baselines have been determined with correctly fixed-integer ambiguities, as shown in Figure 5. The two-way arrow lines indicate that both agents resolved the baseline and DD integer ambiguities. To systematically resolve the remaining integer ambiguities, an agent or central command station generates an integer ambiguity resolution table (ART), as shown in Figure 6. In the ART, the circle ○ indicates agent pairs with correctly fixed DD integer ambiguities. To further resolve the remaining integer ambiguities among the six agents, the pseudocode in Figure 7 can be used.

**Figure 8:**
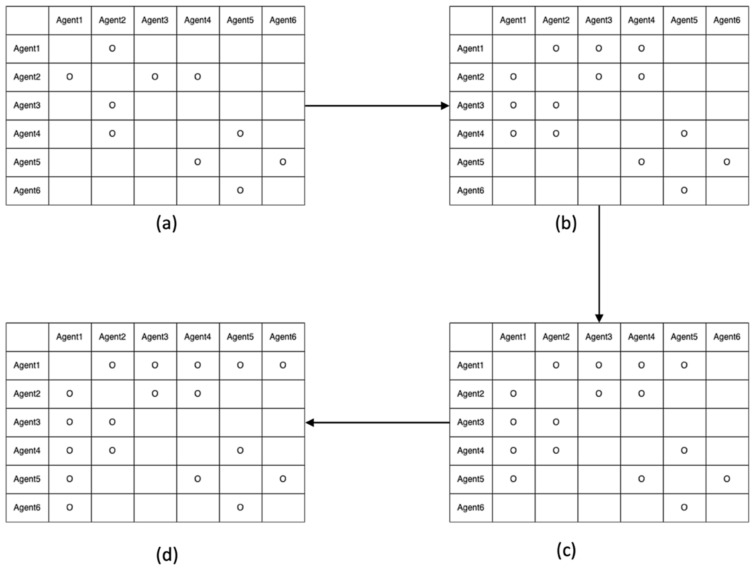
ART processes while implementing the proposed pseudocode. (**a**) Initial ART with resolved integer ambiguities in Figure 5. (**b**) Modified ART by updating $N_{13}$ and $N_{14}$. (**c**) Further modified ART by updating $N_{15}$. (**d**) Further modified ART by updating $N_{16}$.

Using the pseudocode, the integer ambiguities of agent 1 and the other agents in Figure 6 are resolved in the following steps:

(a) Check whether agent 1 resolves integer ambiguities with agent 2. If yes, go to the agent 2 row.
(b) Check if agent 2 resolves integer ambiguities with agents 3 through 6 (i.e., $N_{23}$ and $N_{24}$ would be reported as fixed).
(c) Then, $N_{13}$ and $N_{14}$ are computed using Equation (6).
(d) The ART table is updated, as shown in Figure 8b. Check whether agent 1 resolves the integer ambiguities with agent 3. If yes, go to the agent 3 row.
(e) Agent 3 had no resolved integer ambiguities from agents 4 to 6.
(f) Check whether agent 1 resolves integer ambiguities with agent 4. If yes, go to the agent 4 row.
(g) $N_{45}$ would be reported as fixed.
(h) Then, $N_{15}$ is computed using Equation (6).
(i) Update the ART table as shown in Figure 8c.
(j) Check whether agent 1 resolves integer ambiguities with agent 5. If yes, go to the agent 5 row.
(k) Then, $N_{56}$ would be reported as fixed.
(l) $N_{16}$ is computed using Equation (6).
(m) Update the ART table as shown in Figure 8d.
(n) If the first row has all circles, except for itself, then the procedure terminates.

As shown in Figure 8d, agent 1 resolves the DD integer ambiguities with all the other agents. The remaining agents can resolve the integer ambiguities through agent 1 using Equation (6). In the above procedure, the DD integer ambiguities are assumed to have the same pivot satellite. However, it is possible that some agents may not see the pivot satellites used by the other pairs of agents. This problem can be resolved using a pivot transformation matrix. For example, integer ambiguities with *p* pivot satellites can be transformed into *k* pivot satellites using the following transformation:

(7) $$\left[\begin{array}{c} N^{k1}\\ N^{k2}\\ \vdots \\ N^{kn} \end{array}\right]=\underset{\mathrm{Pivot}\;\mathrm{Transformation}\;\mathrm{Matrix}}{\underset{︸}{\left[\begin{array}{cccccc} 1 & 0 & \cdots & -1 & \cdots & 0\\ 0 & 1 & \cdots & -1 & \cdots & 0\\ \vdots & \vdots & \vdots & \ddots & \cdots & \vdots \\ 0 & 0 & \cdots & -1 & \cdots & 1 \end{array}\right]}}\left[\begin{array}{c} N^{p1}\\ N^{p2}\\ \vdots \\ N^{pk}\\ \vdots \\ N^{pn} \end{array}\right]$$

## 3. Systematic Precise Relative Positioning Using a Cell Structure

Using the antenna array-aided RTK and ART table introduced in the previous section, this section further develops a method to precisely determine the relative position of a swarm of agents by introducing the concept of a cell. The proposed cell structure allows a straightforward implementation of an antenna array-aided RTK and a simpler ART table process than the unstructured case exemplified in the last section.

### 3.1. Cell Structure of Agents

**Figure 9:**
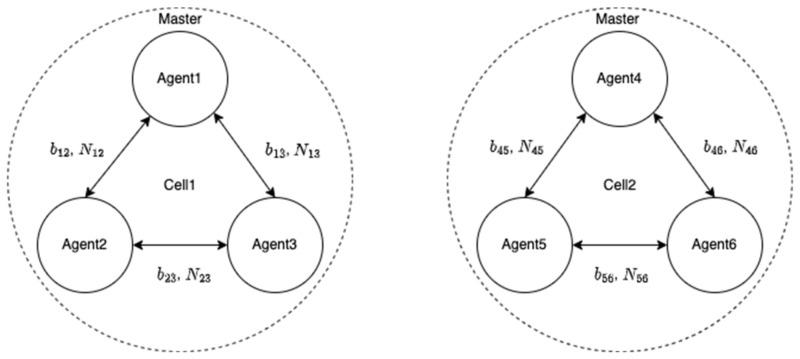
Each cell consists of three agents. One of the agents in each cell is designated as a master agent which is responsible for communication to another cell.

**Figure 10:**
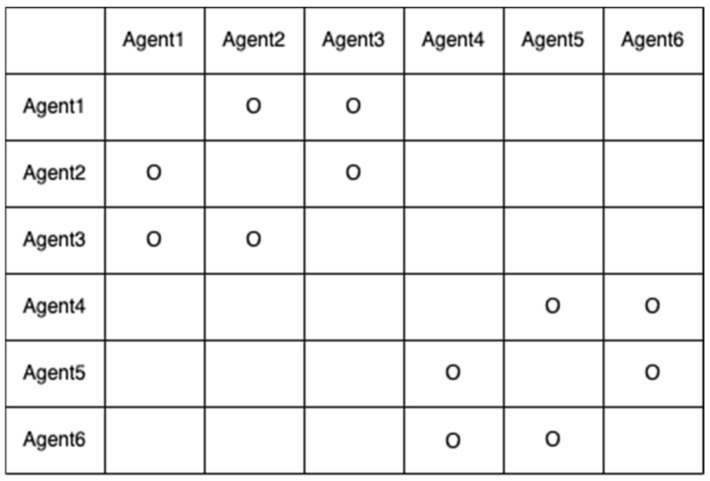
ART table before applying a one-to-one or antenna array-aided RTK across the two cells.

Figure 9 shows the structure of a cell in a group of UVs. Each cell consisted of three agents, and agents 1 and 4 were designated as the master agents in cell 1 and 2, respectively. To establish RTK baselines among all the agents in cell 1, as discussed in the previous section, agents 1 and 2 first implemented a one-to-one RTK. Subsequently, agent 3 executed an antenna array-aided RTK with agent 1 to compute the relative position to agent 1 using Equation (3) and the LAMBDA process. The baseline between agents 2 and 3 can be obtained from the fixed-integer ambiguities of $N_{12}$ and $N_{31}$ using Equation (6). The relative positions in cell 2 can be determined in a similar manner. After obtaining the relative positions in the cell, an ART can be constructed as shown in Figure 10. Notably, the proposed ART process cannot be executed further because there are no established fixed baselines across the two cells.

**Figure 11:**
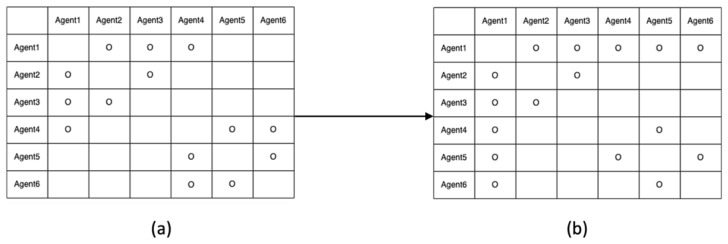
ART table after implementing antenna array-aided RTK with agents 1, 4, and 5. (**a**) Modified ART after the one-to-one RTK between the two master agents. (**b**) Further modified ART by updating $N_{15}$ and $N_{16}$.

In the two cells, agents 1 and 4 acted as the master agents. The role of a master agent is to establish a relative position to the master agent in another cell. Additionally, it delivers the resolved integer ambiguities of the agents within its own cell to the master agent in another cell. Third, the master agent broadcasts the received fixed-integer ambiguities of another cell to the agents in its own cell to obtain the relative position among all agents across the cells. Following these procedures, the corresponding ART process was implemented, as shown in Figure 11. Agent 1 in cell 1 could establish positions relative to all agents in cell 2. Therefore, other agents in the two cells could resolve integer ambiguities through agent 1 and compute the corresponding relative positions.

**Figure 12:**
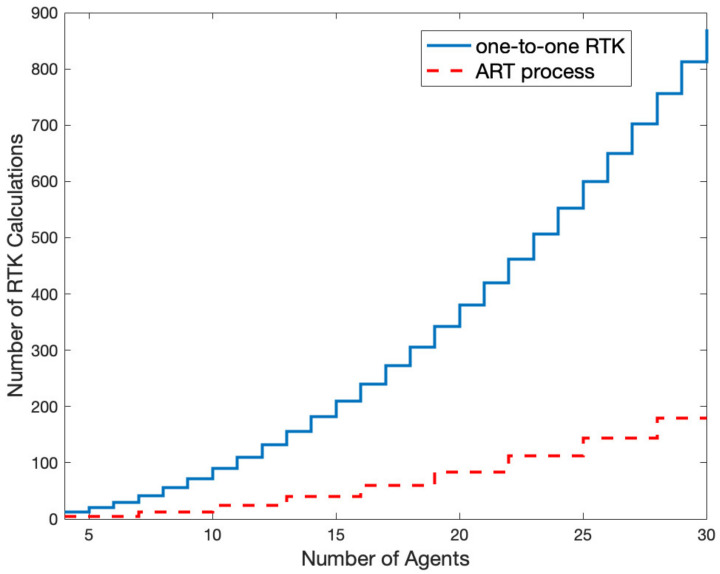
Comparison of the number of computations between one-to-one RTK and ART process with respect to the number of agents.

If a conventional one-to-one RTK process is used to establish fixed baselines among all six agents in the two cells, a total of 30 RTK solutions must be determined. However, the ART process with broadcast-resolved integer ambiguities requires a total of five RTK solutions. Generally, if *n* agents exist, the number of one-to-one RTK computations is $n\times \left(n-1\right)$. With a cell structure consisting of three agents, the number of cells would be $roundup\left(n/3\right)$. Each cell requires at least two RTK computations, and the $roundup\left(n/3\right)$ cells must implement the RTK processes for $roundup\left(n/3\right)\times \left(roundup\left(n/3\right)-1\right)$ times. Therefore, the overall RTK computation required for the ART procedure was $2\times roundup\left(n/3\right)\times \left(roundup\left(n/3\right)-1\right)$. Figure 12 compares the number of RTK computations for the cases of one-to-one RTK and ART.

### 3.2. Proposed Message Fields for ART Process

To implement the antenna array-aided RTK and broadcast-resolved integer ambiguities, the messages in Table 1 should be sent to the other agents.

### 3.3. Fault Monitoring of Broadcast Information

The integrity of the broadcast information is a critical factor for proper outcomes of the ART process. However, it is possible that the broadcast-resolved integer ambiguities and corresponding baseline vectors may have large errors, which would induce biases in the outputs of the ART process. Therefore, fault monitoring should be implemented before initiating the ART.

For the development of reasonable fault monitoring, we consider the difference between the two carrier phase measurements in Equation (3) as follows:

(8) $$T=\left[\begin{array}{ll} I & -I \end{array}\right]\left[\begin{array}{c} \Phi_{32}+\Lambda {\hat{N}}_{12}-G{\hat{b}}_{12}\\ \Phi_{31} \end{array}\right]$$

The elements of the test vector consist of DD carrier phase noise and small position errors if there are no offsets in the resolved DD integer ambiguity of ${\hat{N}}_{12}$ and there are no large biases in ${\hat{b}}_{12}$. The covariance matrix of T is modeled as

(9) $$Cov\left(T\right)=R_{\Phi }+\left[\begin{array}{cc} GR_{b}G^{T} & 0\\ 0 & 0 \end{array}\right]\;$$

where $R_{\Phi }$ and $R_{b}$ are the covariance matrices of the DD carrier phase measurements and baseline solution from a one-to-one RTK, respectively. In this expression, the resolved DD integer ambiguities were treated as constants. The proposed test metric that follows a chi-square distribution can be generated as follows [24,25].

(10) $$v=T^{T}\cdot Cov{\left(T\right)}^{-1}\cdot T\;$$

**Figure 13:**
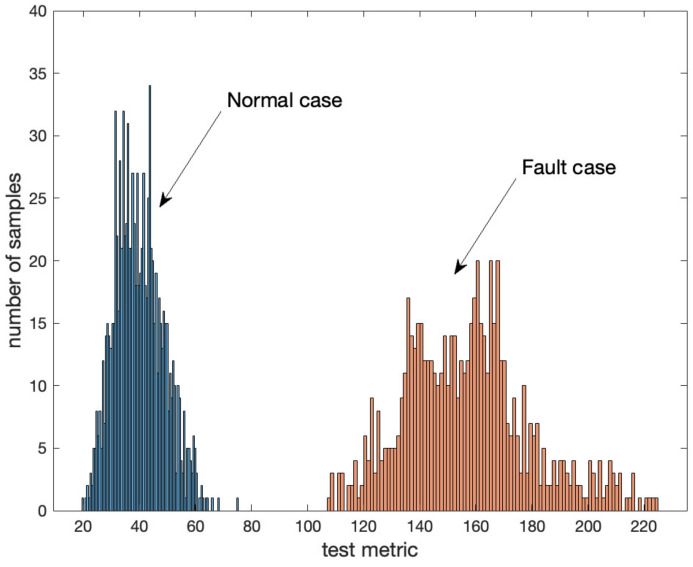
Distributions of v in normal and fault cases based on flight tests.

**Figure 14:**
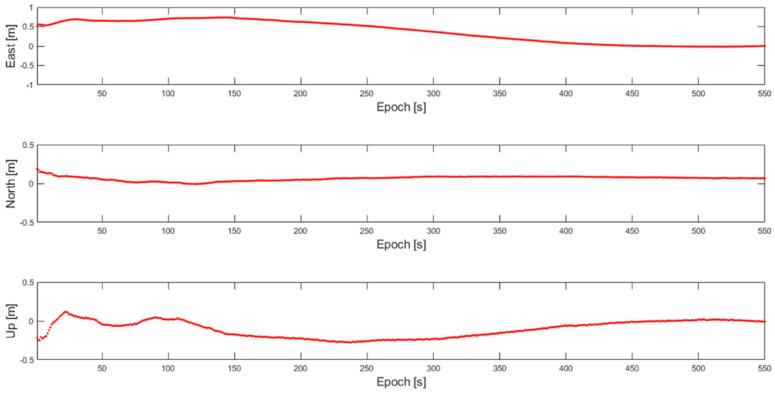
Positioning errors of the broadcast baseline due to injected one cycle offset in one of DD integer ambiguities.

The empirical distribution of v during the flight tests is shown in Figure 13 for the normal and fault cases. There were a total of 1800 samples and each v was computed with 22 measurements. To generate fault measurements, one cycle offset was injected into one of the DD carrier phase measurements, and corresponding positioning errors are shown in Figure 14. Figure 13 shows a large separation between the normal and fault distributions of v. A threshold of v can be determined from a desirable false alarm rate with a central chi-square distribution, and the clear separation of the v distributions in normal and fault cases is expected to allow limited missed detection based on the observed data as shown in Figure 13.

## 4. Numerical Test Results with Multiple GNSS Receiver Measurements

**Figure 15:**
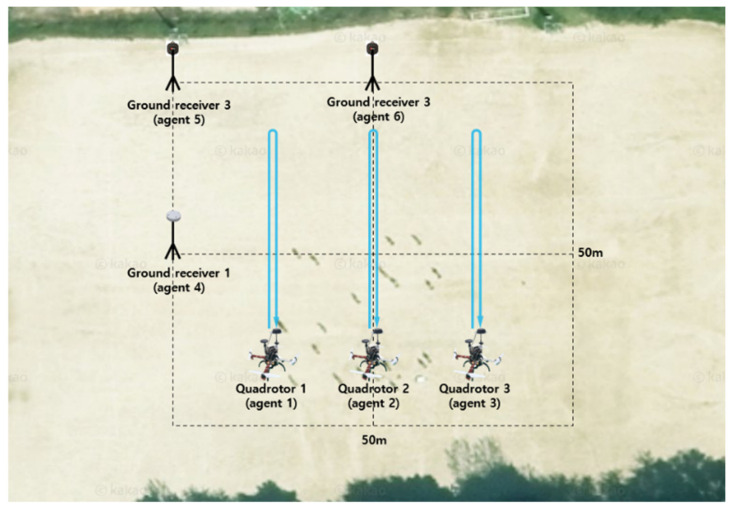
Three quadrotors and GNSS receivers on ground during tests at Hongik University, Seoul, South Korea.

**Figure 16:**
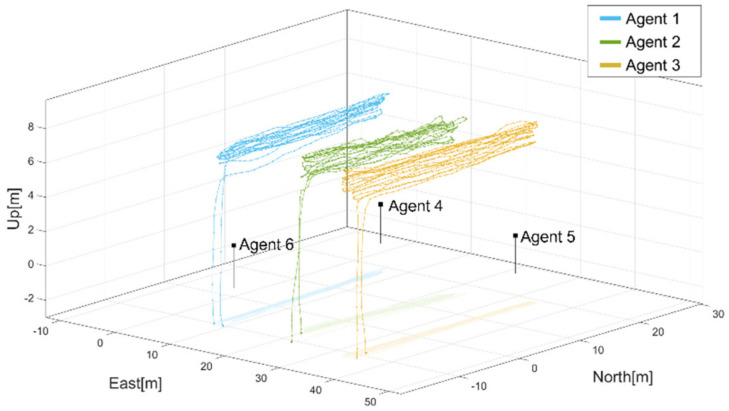
Flight trajectories of agents 1,2, and 3 during the test.

**Figure 17:**
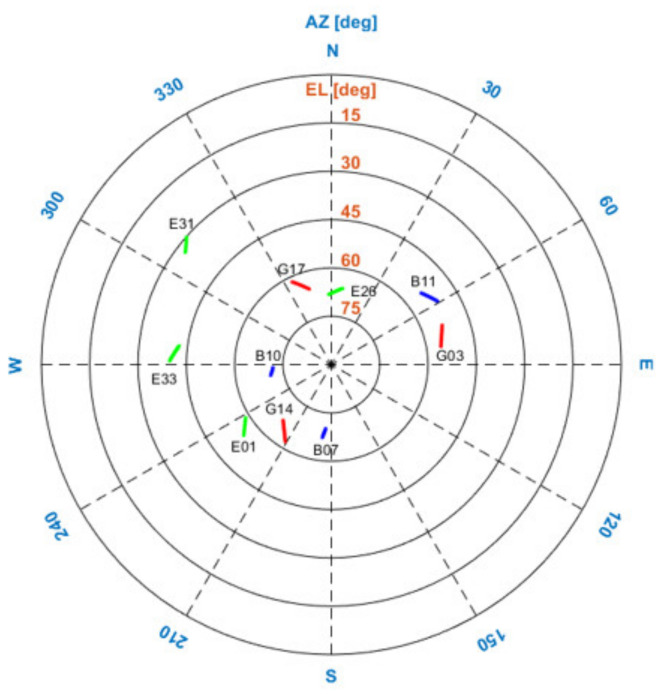
Satellites commonly in view in the two cells during the flight test. GPS, Beidou, and Galileo satellites are indicated by the red, blue, and green lines, respectively.

The proposed ART process was applied to the flight tests. During the flight test, three quadrotors equipped with Ublox F9P GNSS receivers were flying, whereas the other three Novatel GNSS OEM 7700 receivers were statically grounded, as shown in Figure 15. The three quadrotors were designated as agents 1–3, which consisted of cell 1. The three receivers on the ground were designated as agents 4–6, which consisted of cell 2. Figure 16 shows the flight trajectories of agents 1, 2, and 3 in the ENU coordinates. During the test, there were 10 common satellites in view as shown in Figure 17 and dual frequency GNSS measurements were collected at a sampling rate of 1 Hz. Due to the limitation of the quadrotor platform, the speed of the quadrotors was set from 1 to 3 m/s and the flight distance in one way was approximately 30 m. With the chosen sampling rate and speeds of the quadrotors, the one-to-one and antenna array-aided RTK solutions were well converged as will be shown later. All true baselines and DD integer ambiguities were determined from a one-to-one RTK using open-source RTK algorithms [26] and nearby national GNSS reference stations in Korea. The proposed framework was implemented on a computer using collected GNSS measurements in a post-processing mode.

### 4.1. Case of Using all Satellites in Two Cells

**Figure 18:**
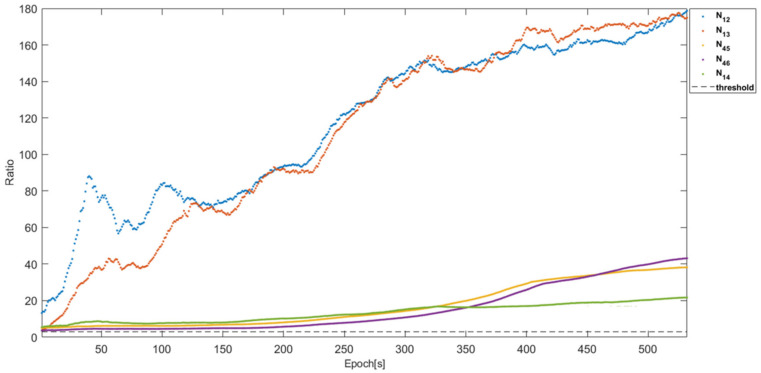
The ratio test results of ***N***_12_, ***N***_13_, ***N***_43_, ***N***_45_, and ***N***_14._ All ratios are greater than three from the first epoch.

**Figure 19:**
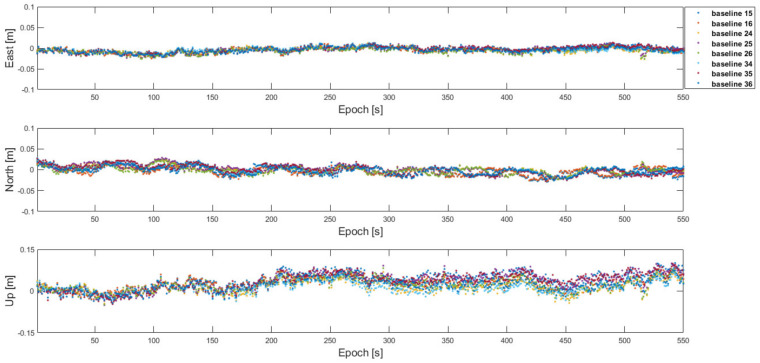
Position errors of the baselines across the two cells using all satellites in view and the broadcast integer ambiguities based on the proposed framework.

Using the proposed RTK framework and flight test data, Table 2 lists the DD integer ambiguities determined from the one-to-one and antenna array-aided RTK using all satellites in view. All integer ambiguities were correctly fixed at the first epoch and did not change during the test, which indicates that the probability of the correctly fixed-integer ambiguities was 100%. The positioning errors in the 3D RMS of the baselines ***b***_12_, ***b***_13_, ***b***_23_, ***b***_43_, ***b***_45_, and ***b***_14_ are listed in Table 3. To show the validity and convergence of the one-to-one and antenna array-aided RTK solutions, Figure 18 shows the ratio test results of ***N***_12_, ***N***_13_, ***N***_43_, ***N***_45_, and ***N***_14_. When the ratios of candidate integer ambiguities are greater than the threshold of three [7], they are usually accepted as correctly resolved integer ambiguities. In Figure 18, the integer ambiguities of ***N***_12_, ***N***_13_, ***N***_43_, ***N***_45_, and ***N***_14_ are greater than three from the first epoch. Notably, ***N***_23_ and ***N***_46_ were computed using Equation (6). Using the proposed framework, the DD integer ambiguities across the two cells were computed using ***N***_14_, as illustrated in Table 4. With the computed DD integer ambiguities, the 3D RMS positioning errors of the other baselines across the two cells were computed, as listed in Table 5. Figure 19 shows the position errors of the baselines in Table 5.

### 4.2. Case of a Degeneate Satellite-in-View Configuration in Two Cells

It is important that the agents in a cell should have a sufficient number of satellites in view to resolve correct integer ambiguities and estimate accurate baselines. Furthermore, a good number of common satellites in view is required between the master agents to implement the one-to-one RTK. However, the number of common satellites in view of the non-master agents between two cells can be small and the proposed framework is still useful to obtain correct DD integer ambiguities across the cells.

**Figure 20:**
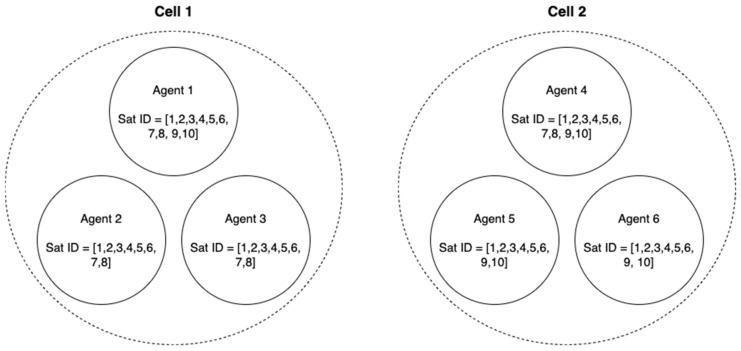
The figure shows satellite IDs that each agent tracked. The two master agents had 10 common satellites and non-master agents had 6 common satellites in view. Each cell had 8 common satellites in view.

**Figure 21:**
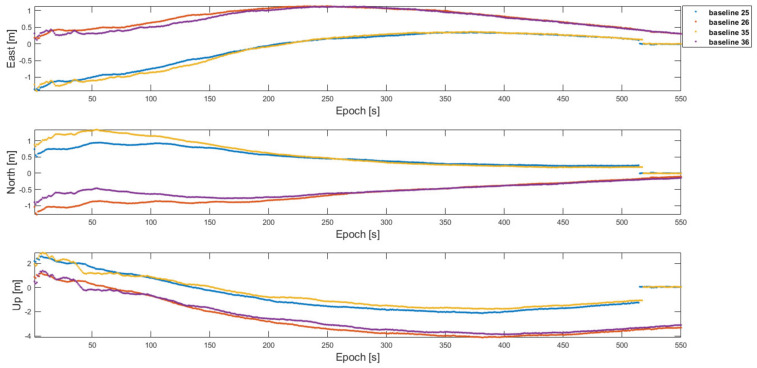
Position errors of the baselines across the cells with the one-to-one RTK using 6 common satellites in view in Figure 20.

To compare the RTK performance between the conventional one-to-one RTK and the proposed framework with a small number of satellites in view, the satellites that the two cells could see during the flight tests were assumed as shown in Figure 20. In this degenerate configuration, the two master agents had 10 common satellites in view, which was a total number of satellites during the flight tests, and each cell had eight common satellites in view. The common satellites in view among the non-master agents between cells 1 and 2 were six satellites. With that, Table 6 lists the fixed 3D RMS errors, overall 3D RMS errors with fixed and float integer ambiguities, and the ambiguity fix success ratio of ***b***_25_, ***b***_26_, ***b***_35_, and ***b***_36_ baselines obtained using the one-to-one RTK with six satellites in view. The ambiguity fix success ratios are very low, and the corresponding positioning errors are large, as presented in Table 6; therefore, the one-to-one RTK cannot be reliably implemented in this case. Figure 21 shows the corresponding position errors of the baselines in Table 6.

**Figure 22:**
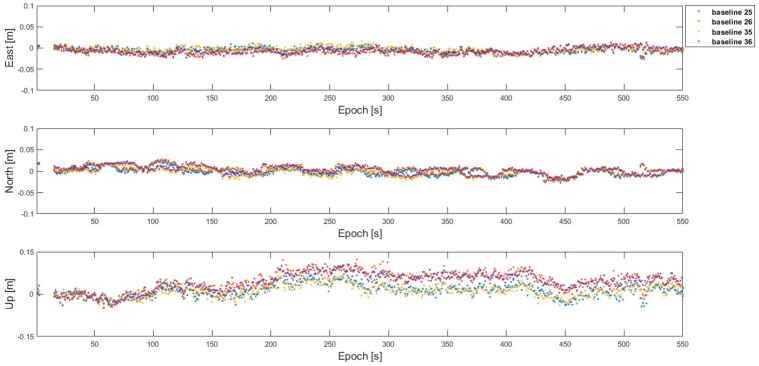
Position errors of the baselines across the cells with the proposed framework using 6 common satellites in view in Figure 20.

To have a better baseline positioning capability across the two cells, the proposed framework was implemented as follows. First, the baseline ***b***_12_, ***b***_13_, ***b***_23_, ***b***_43_, ***b***_45_, ***b***_46_, ***b***_56_, and ***b***_14_ in the two cells were estimated with the one-to-one and antenna array-aided RTK using the satellite configuration in Figure 20. More specifically, ***b***_23_ and ***b***_56_ were first estimated with the one-to-one RTK, respectively. Then, ***b****_1_*_2_ and ***b***_46_ were estimated using an antenna array-aided RTK, respectively. Lastly, ***b***_13_ and ***b***_45_ were estimated using the broadcast fixed ambiguities in each cell, respectively. Table 7 lists the fixed 3D RMS errors, overall 3D RMS errors with fixed and float integer ambiguities, and the ambiguity fix success ratio of the baselines. Because the number of satellites in view in a cell was reduced to eight, the ambiguity fix ratio was less than 100 %. The baselines of ***b***_25_, ***b***_26_, ***b***_35_, and ***b***_36_ across the cells were found by using ***N***_14_ as before and their positioning errors were at the centimeter level as listed in Table 8. Figure 22 shows the corresponding positioning errors of the baselines in Table 8.

In the test results, no faults in the broadcast baselines and integer ambiguities occurred because resolved integer ambiguities passing a ratio test with a threshold of 3 were only used as broadcast baselines [7]. Furthermore, because the proposed framework was implemented on a computer in a post-processing mode, there were no communication errors.

## 5. Conclusions

This study introduced a systematic and efficient GNSS RTK framework for a swarm of moving vehicles, whose applications was exemplified and experimentally tested using six agents. The proposed framework was based on the antenna array-aided RTK and broadcast of fixed-integer ambiguities as well as baselines, which were further manipulated through ART procedures to quickly resolve integer ambiguities among multiple vehicles without resorting to integer ambiguity resolution algorithms, such as LAMBDA. Additionally, a fault-monitoring scheme for broadcast fixed-integer ambiguities and baselines was discussed. Finally, numerical examples of the proposed framework were presented using test data collected from three flying quadrotors and static-ground GNSS receivers. The numerical examples also showed that some agents across cells could have accurate baselines even with six satellites in view when DD integer ambiguities and baselines had been correctly found inside a cell and between master agents. For this case, the conventional one-to-one RTK suffered from a low probability of successful integer ambiguity fix.

While a communication network in the conventional RTK is usually maintained between a rover and a base, a communication network between and within cells should be established for the application of the proposed framework. Such communication networks are also necessary for the control of multiple UVs to accomplish a given mission; therefore, it would be desirable to develop a communication network and message protocols for the positioning and control of multiple UVs. The framework and numerical results proposed in this study can be used for the initial considerations of future RTCM standards for the relative positioning of a swarm of moving vehicles.

## Figures and Tables

**Table 1:** Proposed message fields and descriptions for the ART process.

Message ID	Messages	Description
1	Agent ID	Agent ID broadcasting messages
2	Other agent ID	A vector of agents having resolved integer ambiguities
2	GPS time of week	GPS time of week at the GNSS measurements
3	GNSS satellite PRN numbers	A vector of GNSS PRN number of the resolved integer ambiguities except for the pivot satellites
4	GNSS pivot satellite PRN numbers	A vector of a pivot satellite PRN number corresponding to Agent ID in Message ID 1. It is assumed that there is one pivot satellite per GNSS constellation
5	Fixed-integer ambiguities	A vector of fixed-integer ambiguities corresponding to Agent ID
6	Code phase measurements	A vector of received GNSS code phase measurements in the order of the pivot and other satellites in Message ID 4
7	Carrier phase measurements	A vector of received GNSS carrier phase measurements in the order of the pivot and satellites in Message ID 4
8	Fixed baseline	Estimated fixed baseline to other agents listed in Message ID 1 (this message is not used for a baseline computation but for a sanity check of the broadcast fixed-integer ambiguities)
9	Covariance matrix of fixed baseline	Covariance of the estimated fixed baseline of Message ID8

**Table 2:** DD integer ambiguities within the two cells and between the two master agents using a one-to-one and antenna-array RTK of the proposed framework.

Integer Index	Cell 1			Cell 2			Inter-Cells
	*N*_12_	*N*_13_	*N*_23_	*N*_45_	*N*_46_	*N*_56_	*N*_14_
1	−2	−17	−15	−7	−8	−1	−7
2	0	2	2	6	13	7	−12
3	−12	−3	9	10	16	6	−20
4	7	6	−1	3	−3	−6	5
5	−16	−2	14	15	21	6	−17
6	−13	−2	15	5	2	−3	−7
7	−11	−7	4	0	6	6	−4
8	−7	−11	−4	−7	−4	3	−10
9	5	13	8	−7	−20	−13	−7
10	−2	4	6	0	1	1	−15
11	−5	−2	3	−13	−15	−2	12
12	−5	1	6	−5	0	5	1
13	−2	−7	−5	−8	−7	1	3
14	−9	−1	8	−1	3	4	1

**Table 3:** 3D RMS positioning errors of the baselines in the two cells and two master agents using a one-to-one and antenna-array RTK of the proposed framework.

	Cell 1			Cell 2			Inter-Cells
	*b*_12_	*b*_13_	*b*_23_	*b*_45_	*b*_46_	*b*_56_	*b*_14_
3D RMS (cm)	1.79	1.72	1.63	1.91	1.92	0.66	2.94

**Table 4:** DD integer ambiguities across the two cells using the broadcast integer ambiguities of the proposed framework.

	*N*_15_	*N*_16_	*N*_24_	*N*_25_	*N*_26_	*N*_34_	*N*_35_	*N*_36_
1	−20	−28	−5	−18	−26	10	−3	−11
2	−18	−25	−12	−18	−25	−14	−20	−27
3	−10	−4	−8	2	8	−17	−7	−1
4	8	2	−2	1	−5	−1	2	−4
5	−2	4	−1	14	20	−15	0	6
6	−2	−5	6	11	8	−9	−4	−7
7	−4	2	7	7	13	3	3	9
8	−10	6	−3	−3	13	1	1	17
9	0	13	−12	−5	8	−20	−13	0
10	−15	−14	−13	−13	−12	−19	−19	−18
11	−1	−3	17	4	2	14	1	−1
12	−4	1	6	1	6	0	−5	0
13	−5	−4	5	−3	−2	10	2	3
14	0	4	10	9	13	2	1	5

**Table 5:** The 3D RMS position errors of the baselines across the two cells using all satellites in view and the broadcast integer ambiguities of the proposed framework.

	*b*_15_	*b*_16_	*b*_24_	*b*_25_	*b*_26_	*b*_34_	*b*_35_	*b*_36_
3D RMS (cm)	5.04	3.69	2.88	4.75	3.43	2.72	4.60	3.25

**Table 6:** DD integer ambiguity fix success probabilities and 3D RMS position errors of the baselines across the two cells using a one-to-one RTK with six common satellites in view in Figure 20.

	*b*_25_	*b*_26_	*b*_35_	*b*_36_
Fixed 3D RMS (cm)	5.15	NA	5.31	NA
Overall 3D RMS (cm)	169.57	332.60	158.62	303.80
Ambiguity fix success probability (%)	6.55	0	6.00	0

**Table 7:** The 3D RMS positioning errors of the baselines in the two cells and two master agents using a one-to-one and antenna-array RTK of the proposed framework with the satellite configuration in Figure 19.

	Cell 1			Cell 2			Inter-Cells
	*b*_12_	*b*_13_	*b*_23_	*b*_45_	*b*_46_	*b*_56_	*b*_14_
Fixed 3D RMS (cm)	1.98	1.92	1.74	1.77	1.77	1.90	2.94
Overall 3D RMS (cm)	5.10	N/A	95.1	45.0	N/A	28.1	2.94
Ambiguity fix success probability (%)	99.8	N/A	83.1	96.1	N/A	98.7	100

**Table 8:** 3D RMS positioning errors of the baselines across the two cells with the proposed framework using 6 common satellites in view in Figure 19.

	*b*_25_	*b*_26_	*b*_35_	*b*_36_
3D RMS (cm)	5.29	3.23	5.12	3.06
